# Combining sequencing approaches to fully resolve a carbapenemase-encoding megaplasmid in a *Pseudomonas shirazica* clinical strain

**DOI:** 10.1080/22221751.2019.1648182

**Published:** 2019-08-05

**Authors:** João Botelho, Cédric Lood, Sally R. Partridge, Vera van Noort, Rob Lavigne, Filipa Grosso, Luísa Peixe

**Affiliations:** a UCIBIO/REQUIMTE, Laboratório de Microbiologia, Faculdade de Farmácia, Universidade do Porto, Porto, Portugal; b Centre of Microbial and Plant Genetics, Department of Microbial and Molecular Systems, KU Leuven, Leuven, Belgium; c Laboratory of Gene Technology, Department of Biosystems, KU Leuven, Leuven, Belgium; d Centre for Microbiology and Infectious Diseases, The Westmead Institute for Medical Research, The University of Sydney, Westmead Hospital, Sydney, Australia; e Institute of Biology, Leiden University, Leiden, The Netherlands

**Keywords:** *Pseudomonas*, megaplasmids, Nanopore, Illumina, antibiotic resistance

## Abstract

Horizontal transfer of plasmids plays a pivotal role in dissemination of antibiotic resistance genes and emergence of multidrug-resistant bacteria. Plasmid sequencing is thus paramount for accurate epidemiological tracking in hospitals and routine surveillance. Combining Nanopore and Illumina sequencing allowed full assembly of a carbapenemase-encoding megaplasmid carried by multidrug-resistant clinical isolate FFUP_PS_41. Average nucleotide identity analyses revealed that FFUP_PS_41 belongs to the recently proposed new species Pseudomonas shirazica, related to the P. putida phylogenetic group. FFUP_PS_41 harbours a 498,516-bp megaplasmid (pJBCL41) with limited similarity to publicly-available plasmids. pJBCL41 contains genes predicted to encode replication, conjugation, partitioning and maintenance functions and heavy metal resistance. The |aacA7|blaVIM-2|aacA4| cassette array (resistance to carbapenems and aminoglycosides) is located within a class 1 integron that is a defective Tn402 derivative. This transposon lies within a 50,273-bp region bound by Tn3-family 38-bp inverted repeats and flanked by 5-bp direct repeats (DR) that composes additional transposon fragments, five insertion sequences and a Tn3-Derived Inverted-Repeat Miniature Element. The hybrid Nanopore/Illumina approach allowed full resolution of a carbapenemase-encoding megaplasmid from P. shirazica. Identification of novel megaplasmids sheds new light on the evolutionary effects of gene transfer and the selective forces driving antibiotic resistance.

## Introduction

Bacteria can become resistant to antibiotics through chromosomal mutations and/or by the acquisition of resistance genes carried on mobile genetic elements, including plasmids and integrative and conjugative elements [1]. Plasmids are autonomous self-replicating elements of which some are capable to drive horizontal transfer (HGT) of antibiotic resistance genes by conjugation [2–5]. The mobility of a plasmid depends on the set of genes that it carries, and these extrachromosomal elements may be conjugative, mobilisable or non-transmissible [2,3]. Conjugative plasmids carry all the machinery necessary for self-transfer: i) a relaxase, a key protein in conjugation; ii) an origin of transfer (*oriT*); iii) a set of genes encoding for the type-IV secretion system (T4SS); and iv) a gene encoding a type-IV coupling protein (T4CP) [2,3]. Mobilisable plasmids lack the complete set of genes encoding the T4SS and may use the conjugative apparatus of a helper plasmid present in the cell to be successfully transferred. Conjugative plasmids tend to be low copy number and large, whereas mobilisable plasmids are frequently high copy number and smaller (<30 kb) [2,3]. The term megaplasmids [6] has been used for very large replicons (>350 kb) which, in contrast to chromids [7], do not carry essential core genes. Megaplasmids frequently have mosaic structures, carrying genetic modules that originate from different ancestral sources [8]. The formation of mosaic plasmids may be influenced by several factors, such as the abundance of conjugative plasmids and transposons, selection pressures, incompatibility groups and the host’s tolerance of foreign DNA. According to the plasmid hypothesis, megaplasmids are the evolutionary precursors of chromids, due to the amelioration of genomic signatures to those of the host’s chromosome and the acquisition of essential genes [7].

To date, fourteen incompatibility groups (IncP-1 to IncP-14) have been characterised amongst *Pseudomonas* plasmids [9,10]. Narrow host range plasmids comprise IncP types -2, -5, -7, -10, -12 and -13 and cannot be transferred into *Escherichia coli*. In contrast, other groups display a broad host range, as they are also included in the typing scheme for *Enterobacteriaceae* plasmids: IncP-1 (IncP), IncP-4 (IncQ) and IncP-6 (IncG) [9,10]. Unlike *Enterobacteriaceae* plasmids, no replicon-based PCR typing of other *Pseudomonas* plasmids has been created yet. Even though a few reports have characterised large plasmids among pseudomonads [11–13], the role of these elements in the spread of antibiotic resistance in this genus remains poorly understood.

Plasmids may harbour accessory module(s) that provide adaptive advantage(s) for their host, such as virulence-encoding factors and antibiotic resistance genes [9,14–16]. These elements frequently harbour carbapenemase-encoding genes, which confer resistance to β-lactams, including carbapenems, frequently last resort antibiotics for infections caused by multi-drug resistant bacteria [9,17]. Sequencing of plasmids is thus paramount to the success of accurate epidemiological tracking strategies in the hospital setting and routine surveillance, helping to identify transmission routes and to prevent future outbreaks [18–23]. The advent of WGS has enabled the *in silico* analysis of a wide array of plasmids, most of them from assembly of short-read sequencing data [11,24–27]. However, fully resolving plasmids with short-read sequencing technologies remains challenging due to the presence of numerous long repeated regions [28], and currently the most accurate approach to assemble these plasmids is to use a combination of short-read and long-read methods [18–23,29,30].

Here, we combined Nanopore and Illumina sequencing to fully assemble a carbapenemase-encoding megaplasmid carried by a clinical isolate belonging to the recently proposed *Pseudomonas shirazica* species [31].

## Material and methods

### Bacterial isolate

Isolate FFUP_PS_41 was obtained in 2008 from endotracheal tube secretions of a patient with pneumonia admitted to the Neonatal/Pediatric Intensive Care unit of Centro Hospitalar do Porto – Hospital de Santo António, in Porto, Portugal, as part of regular surveillance of carbapenemase-producers among clinical isolates.

FFUP_PS_41 was initially identified at the hospital as *Pseudomonas putida* by VITEK-2 (bioMérieux), a routine phenotypic based method for bacterial identification. In this study we re-classified the strain by pair-wise average nucleotide identity based on BLAST+ (ANIb) using PyANI v0.2.7 (https://github.com/widdowquinn/pyani) [32,33]. Antimicrobial susceptibility testing was conducted by standard disc diffusion and broth microdilution (for colistin) methods, according to EUCAST guidelines (http://www.eucast.org/).

### Whole-plasmid sequencing and bioinformatics

Genomic DNA from FFUP_PS_41 was extracted using a QIAamp DNA Mini Kit (Qiagen, Hilden, Germany) according to the manufacturer’s instructions. Sequencing libraries were prepared using Illumina Nextera and the 1D ligation library approach from Oxford Nanopore Technology (ONT) where we used a Covaris gTube to fragment the gDNA around 10 kbp. Libraries were sequenced on the Illumina HiSeq 2500 sequencer or the MinION sequencer from ONT equipped with a flowcell of chemistry type R9.4, respectively.

Illumina reads were verified for quality using FastQC and Trimmomatic [34,35] yielding 5.9 M paired-end reads of 125 bp in length totalling 1.4 B bases (245x est. coverage), while the MinION reads were processed with ONT’s albacore v2.3.0 followed by demultiplexing using porechop v0.2.3, yieding 62.6 k reads totalling 504 M bases (84x est. coverage). Both datasets were then combined using the Unicycler assembly pipeline [36] with a finishing step of Pilon v1.22. The assemblies were visually inspected using the assembly graph tool Bandage v0.8.1 [37]. Annotation of the megaplasmid was performed with Prokka v1.13 using default parameters [38]. To improve annotation, we downloaded additional files of trusted proteins from NCBI RefSeq plasmids (ftp://ftp.ncbi.nih.gov/refseq/release/plasmid/), the NCBI Bacterial Antimicrobial Resistance Reference Gene Database (ftp://ftp.ncbi.nlm.nih.gov/pathogen/Antimicrobial_resistance/) and the Antibacterial Biocide- and Metal-Resistance Genes database (Bac-Met, http://bacmet.biomedicine.gu.se/index.html, all accessed on the 01/10/18). EggNOG mapper v4.5.1 and NCBI's Conserved Domain Database CDSEARCH/cdd v3.16 were used for functional annotation and conserved domain search of protein sequences, respectively [39–41]. Inference of orthologous groups (OGs) was achieved with OrthoFinder v2.2.6 [42]. The coding sequence (CDS) annotations of the megaplasmid were visualised with Circos v0.69–6 [43]. We used ISfinder [44] to look for insertion sequences (IS). Antimicrobial resistance genes and associated mobile elements were annotated using Galileo^TM^ AMR (https://galileoamr.arcbio.com/mara/, Arc Bio, Cambridge, MA) [45]. Plasmid copy number was estimated based on coverage of the Illumina dataset. GenSkew (http://genskew.csb.univie.ac.at/) was used to compute and plot nucleotide skew data to predict the origin of replication.

### Plasmid transfer and S1/ICeuI-PFGE

Plasmid transfer by conjugation assays was attempted as previously described [46], using a spontaneous rifampicin-resistant mutant of *P. aeruginosa* PAO1 as recipient strain. Transconjugant selection was performed using Mueller–Hinton agar containing rifampicin (100  mg/L) and imipenem (2  mg/L).

S1 and I-CeuI-PFGE was performed as previously described [47] to confirm the presence of extrachromosomal elements.

### Accession number

The sequence of plasmid pJBCL41 was deposited in GenBank accession number MK496050.

## Results

### Antimicrobial susceptibility and taxonomy testing

Clinical isolate FFUP_PS_41 has a multidrug resistance (MDR) phenotype, showing resistance to imipenem, meropenem, ceftazidime, cefepime, aztreonam, piperacilin + tazobactam, gentamicin, tobramycin, amikacin, ciprofloxacin but remains susceptible to colistin (MIC = 1 mg/L). FFUP_PS_41 was initially identified as *P. putida* by VITEK-2. However, it displays an ANIb value of 99.1% (above the 95% cut-off for species identification [32]) when compared with the *P. shirazica* type strain genome [31], suggesting that it belongs to this species related to the *P. putida* phylogenetic group.

### Comparative megaplasmidomics between pJBCL41 and related Pseudomonas plasmids

**Figure 1.**
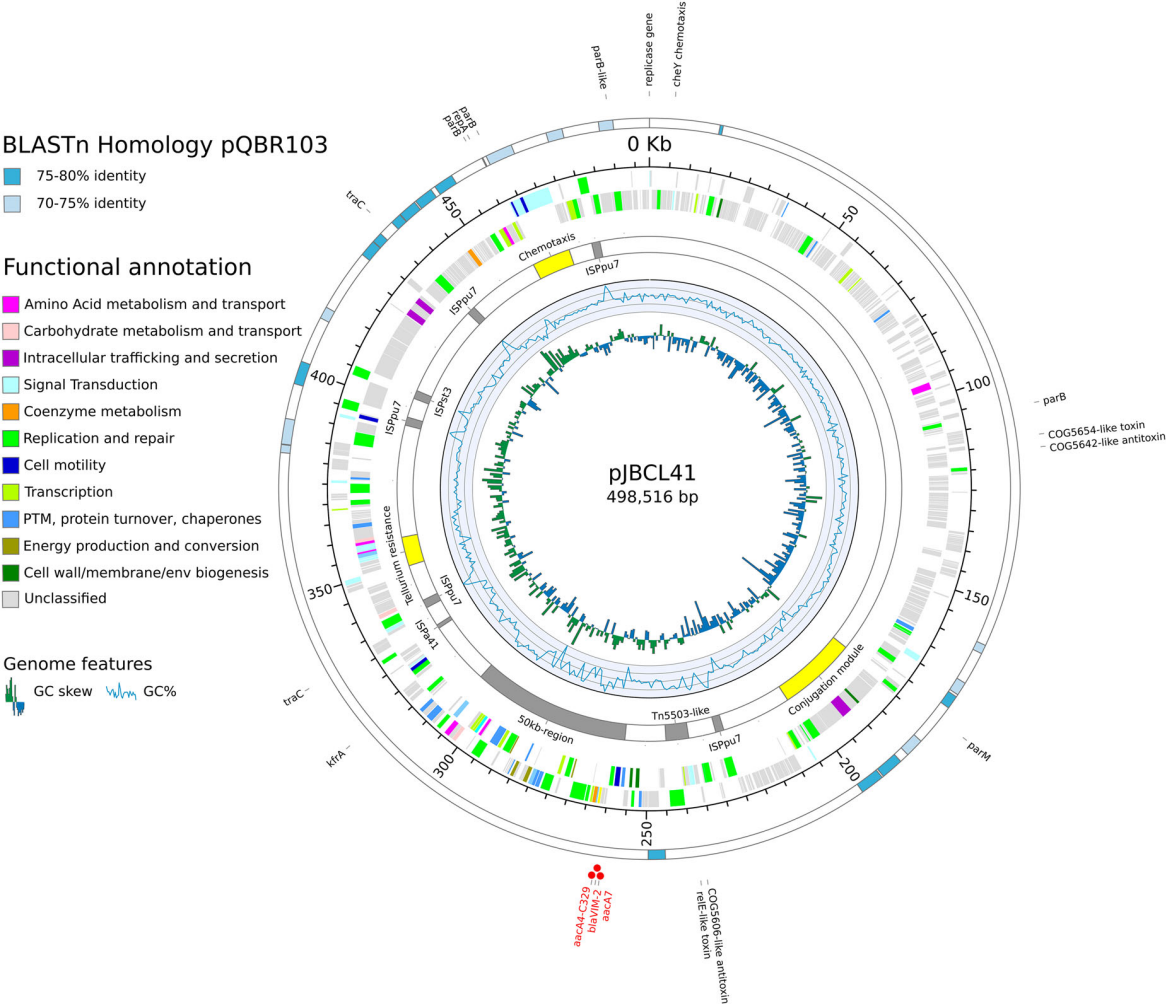
Circular representation of genomic features of pJBCL41. The innermost circle is a histogram of the GC skew, the next a graph of GC content. The next circle displays selected regions of interest (yellow) and IS and transposons or related elements (grey). The next two circles represent the coding regions on the negative and positive strands coloured by their functional annotation (when available). The outermost circle displays regions with high levels of identity to pQBR103 (GenBank accession no. NC_009444.1). Red dots highlight genes encoding antibiotic resistance.

Using a hybrid assembly approach, we were able to fully resolve a single extrachromosomal element carried by *Pseudomonas* sp. FFUP_PS_41 (Figure S1). This mosaic megaplasmid (named pJBCL41) is 498,516 bp long and a total of 608 predicted CDS were annotated (Figure 1). It has an average GC content of 56.0%, which is lower than that observed for the chromosome (62.6%) and the mean content for strains identified as *P. putida* (62.0%, according to information retrieved on the 08/03/2019 on https://www.ezbiocloud.net/taxon?tn=Pseudomonas%20putida).

**Figure 2.**
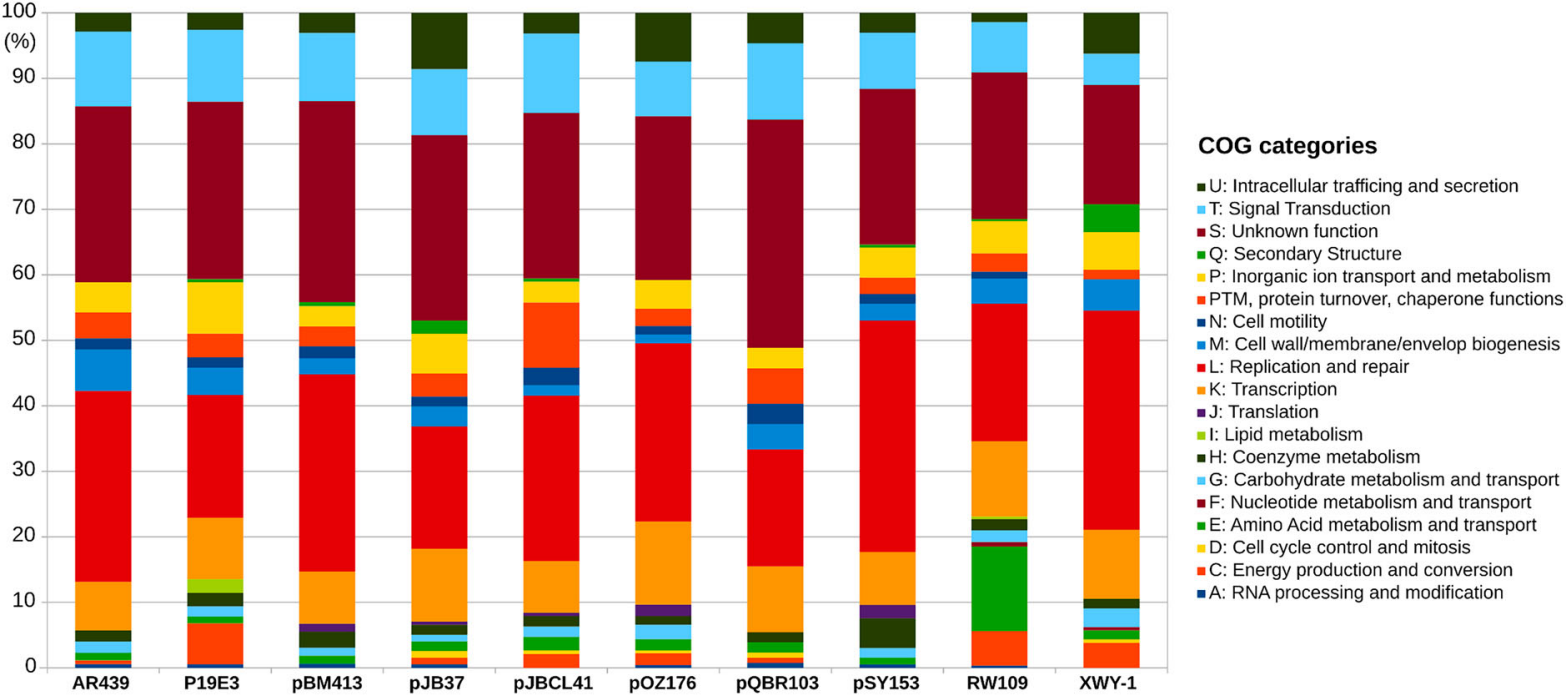
Functional characterization of pJBCL41 and related megaplasmids. COG stands for Cluster of Orthologous Groups.

NCBI’s conserved domains database (CDD) calls 42.1% (256) of the predicted CDS for pJBCL41 (Table S1), indicating that most genes encode proteins of unknown function. The backbone of this megaplasmid harbours genes predicted to be responsible for plasmid replication and heavy metal resistance and carries two predicted type-II toxin-antitoxin (TA) systems and genes encoding for partition systems (Figure 1) [48]. Several genes encoding transport and metabolic processes, as well as transposable elements and CDS associated with transcription, regulatory, chemotaxis signal transduction and mobility functions could be identified (Table S2). These traits are frequently overrepresented on large plasmids (Figure 2) [6,49]. Also, pJBCL41 harbours several genes coding for the synthesis of DNA precursors, which may promote replication and transcription processes to help alleviate the burden that this acquired element may impose on the host cell.

pJBCL41 has low nucleotide sequence identity with *Pseudomonas* megaplasmids deposited in public databases (Table 1 and Figure S2). OrthoFinder assigned 59.4% of proteins encoded by pJBCL41 and the most closely-related plasmid, pQBR103 from *Pseudomonas fluorescens* [50], to 335 OGs (Table S3). pQBR103 was found in *Pseudomonas* populations colonising the leaf and root surfaces of sugar beet plants growing at Wytham, United Kingdom and carries no antimicrobial resistance genes [50]. Curiously, a blastp analysis between the proteins encoded by these megaplasmids revealed that the average amino acid sequence identity is 72.8% among sequences producing significant alignments.

**Table 1.** Megaplasmids identified by BLASTn search with pJBCL41.

Plasmid identifier	Max score	Total score	Query cover	E value	Ident (%)	Species	Genbank accession	Size (bp)	Year of isolation	Source	Country
pQBR103	11073	1.06E + 05	44%	0	72.99%	*P. fluorescens*	NC_009444.1	425094	2008	Environment	UK
XWY-1	10059	1.22E + 05	9%	0	99.59%	*P. sp.*	NZ_CP026333.1	394537	2016	Environment	China
pJB37	7285	43666	17%	0	99.90%	*P. aeruginosa*	KY494864.1	464804	2008	Clinical	Portugal
pSY153	6131	1.41E + 05	20%	0	99.85%	*P. putida*	KY883660.1	468170	2012	Clinical	China
pOZ176	6129	77698	19%	0	99.71%	*P. aeruginosa*	KC543497.1	500839	2000	Clinical	China
pBM413	5306	51713	17%	0	99.93%	*P. aeruginosa*	CP016215.1	423017	2012	Clinical	China
RW109	4728	41531	17%	0	71.75%	*P. aeruginosa*	NZ_LT969519.1	555265	NA	Industrial	NA
P19E3	4715	28494	15%	0	71.79%	*P. koreensis*	NZ_CP027478.1	467568	2014	Environment	Switzerland
AR439	4697	39223	16%	0	71.79%	*P. aeruginosa*	NZ_CP029096.1	437392	NA	Clinical	NA

NA indicates no data available.

Large plasmids identified among the *Pseudomonas* genus usually belong to the IncP-2 incompatibility group [10,11,27]. However, the IncP-2-type stability/replication/conjugal transfer system is absent from pJBCL41 as previously observed for other megaplasmids carried by different *Pseudomonas* species [51,52]. Two replication initiation genes could be identified here. One replicase gene is located at positions 458,679–457,813 on the plasmid (locus_tag: pJBCL41_00568), in close proximity to the predicted origin of replication (Figure S3). pJBCL41 is estimated to be present as a single copy, from read coverage vs. the chromosome. Like many megaplasmids, pJBCL41 appears to possess a full set of genes for self-transmission [2,3]. We identified a cluster of genes encoding an F-type T4SS, encompassing i) a gene encoding a TraD homolog (locus_tag: pJBCL41_00295), an AAA+ ATPase of the pfamVirD4 type, known as the T4CP and which is a key protein in conjugation; ii) a gene encoding a TraI (locus_tag: pJBCL41_00297) relaxase homolog, which together with accessory proteins is responsible for cleaving the plasmid in a site-specific manner to initiate DNA transfer and iii) a set of genes (*traEFGKNV* homologues, positions 182,497–203,751) coding for a mating pair formation system responsible for pilus assembly and retraction (Figure 1) [2,3,53].

We were unable to transfer the pJBCL41 *in vitro* to a spontaneous rifampicin-resistant mutant of *P. aeruginosa* PAO1, under tested conditions. S1/I-CeuI-PFGE confirmed the presence of a ∼500 kb extrachromosomal element.

### pJBCL41 carries a complex 50 kb multidrug resistance region

**Figure 3.**
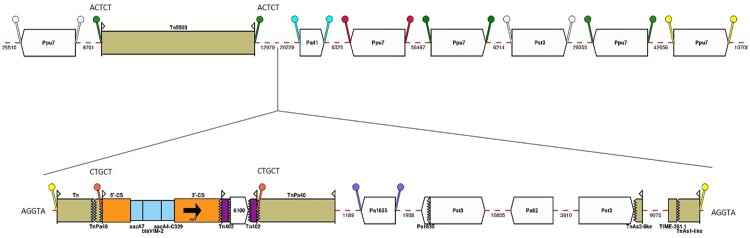
Map of resistance genes and mobile genetic elements inserted in the backbone of pJBLC41. Gene cassettes are shown as blue boxes labelled with the cassette name and are oriented in the 5'-CS to 3'-CS direction. IS are shown as block arrows labelled with the IS name/number, with the pointed end corresponding to IR_R_. TIME-261.1 and fragments of Tn*3*-family transpospons are shown as beige boxes with 38 bp IR represented by flags. The fragment annotated as “TnAs1-like” is ∼97% identical to a region in common between Tn*1721* (GenBank accession no. X61367.1) and Tn*As1* in ISfinder. The fragment annotated as “TnAs2-like” is ∼94% identical to Tn*As2* in ISfinder. The integron is inserted in a proposed hybrid transposon, apparently created by *res*-mediated recombination between a *tnp* region matching Tn*Pa40* and another tranpsoson, labelled “Tn”, that is ∼86% identical to Tn*As1* over the ∼300 bp at the IR_L_ end only. DR are shown as a pair of “lollipops” of the same colour flanking an IS or a pair of IRs (but note that the same colour may be used to indictate more than one pair of DR), with sequences indicated for DR of transposons. Mobile elements are shown to scale and numbers below dashed red lines indicate the lengths of intervening regions in bp. This figure was constructed from diagrams generated using Galileo^TM^ AMR.

pJBCL41 carries genes typically found on IncP-2 plasmids encoding resistance to tellurite, which could allow co-selection and enrichment of bacteria with MDR plasmids [54]. It also harbours a class 1 integron with the |*aacA7*|*bla*_VIM-2_|*aacA4*| cassette array (named In103 by INTEGRALL [55]) (Figure 3): *aacA7* confers resistance to aminoglycosides (amikacin, netilmicin and tobramycin) and *bla*_VIM-2_ encodes resistance to β-lactams (including carbapenems). The *bla*_VIM-2_ gene is by far the most frequently described carbapenemase-encoding gene, both geographically and phylogenetically (across *Pseudomonas* spp.) [56,57]. The *aacA4* gene cassette has a C residue at nucleotide position 329 corresponding to a serine residue associated with gentamicin resistance [58]. The same cassette array has been observed previously among isolates from Portuguese hospitals [25]. The integron is of the In4 type, with a complete 5′-CS bounded by the 25 bp inverted repeat IRi, 2,239 bp of the 3′-CS and IS*6100* flanked by two fragments of the IRt end of Tn*402* [9,59]. As the region between IRi and IRt lacks *tni* transposition genes, this constitutes a Tn*402*-like transposon that would be defective in self-transposition.

This defective Tn*402*-like transposon is flanked by 5-bp direct repeats (DR) (5´-CTGCT-3´) (Figure 3), suggesting integration by transposition close to the predicted resolution (*res*) site of a Tn*3*-family transposon. About 300 bp at the IR_L_ end of the transposon are related (∼86% identical) to Tn*As1* (ISfinder), followed by a region containing a gene which may encode a methyl-accepting chemotaxis protein. From the predicted recombination crossover point in the *res* site the sequence matches Tn*Pa40* (ISfinder). This “hybrid” transposon is not flanked by characteristic 5 bp DR but the 5 bp adjacent to IR_L_ (5´-AGGTA-3´) are repeated 50,273 bp away, immediately adjacent to the 38 bp repeat of a 1,100 bp transposon fragment ∼97% identical to part of both Tn*1721* (GenBank accession no. X61367.1, [60]) and Tn*As1* (Figure 3). This transposon is truncated by 261 bp region that apparently corresponds to a Tn*3*-Derived Inverted-Repeat Miniature Element (designated TIME-261.1 here). TIMEs are non-autonomous mobile elements commonly found in *Pseudomonas* spp. [61].

Most of the region between these transposon elements consists of a 16,782 bp segment flanked by directly oriented copies of IS*Pst3* (IS*21* family). This region, except for insertion of IS*Pa82* (IS*66* family) and an adjacent deletion in pJBCL41, matches several *Pseudomonas* chromosomes (e.g. *P. aeruginosa* PA7 in Figure S4) and different parts of it are found in plasmids in *Pseudomonas*, *Acinetobacter* and *Enterobacteriaceae*, sometimes also flanked by IS. The sequence between Tn*Pa40* and the left-hand IS*Pst3* in pJBCL41 is a duplication of part of the 16,782 bp region, with IS*Pa1635* (IS*4* family) inserted, flanked by characteristic 8 bp DR, instead of IS*Pa82* and ends with a partial IS*Pa1635*. The right-hand IS*Pst3* truncates a transposon related to Tn*As2* [62], which is separated from TIME-261.1 by a 9,075 bp region that also matches *Pseudomonas* chromosomes and includes a putative aminoglycoside phosphotransferase gene.

Blast searches with the complete 50 kb region identified a 59 kb region in the chromosome of *P. aeruginosa* AR_0440 (GenBank accession no. CP029148.1) that has similar ends, but lacks an integron, with an additional Tn*5393* insertion and a different region in place of the IS*Pst3*-bounded segment (Figure S4). This 59 kb region is flanked by 5 bp DR (5´-AATGA-3´) and an uninterrupted version of the flanking sequence matches other *Pseudomonas* chromosomes.

A Tn*5503*-like transposon encoding a type-II TA system and two metal dependent phosphohydrolases is also inserted in pJBCL41 [63] and is flanked by 5-bp DR (5´-ACTCT-3´), indicating that this element transposed independently of the 50 kb region (Figure 3). It has only 10 nucleotide differences from the original Tn*5503* on plasmid Rms149, the archetype of *Pseudomonas* plasmid incompatibility group IncP-6 [63], and additional copies of short repeats in a GC-rich region within a gene encoding an ATP-utilizing enzyme. An additional IS*Pst3*, five IS*Ppu7* (IS*21* family) and one IS*Pa41* (IS5 family) - all flanked by DR of characteristic length, are also inserted in the pJBCL41 backbone (Figures 1 and 3).

## Discussion

In this study, we took advantage of a hybrid assembly approach to fully resolve and characterise a carbapenemase-encoding megaplasmid and to compare it with related *Pseudomonas* megaplasmids. The lower GC content of pJBCL41 compared with the FFUP_PS_41 chromosome and strains belonging to the *P. putida* phylogenetic group may be related to a more relaxed selection acting on these secondary replicons, as the maintenance of GC-rich genomes is energetically more demanding [64,65]. Ongoing studies will help to characterise the biology and genomic signatures related to this newly characterised *P. shirazica* species (Botelho *et al*, unpublished data). Even though we were unable to transfer pJBCL41 by conjugation to a *P. aeruginosa* strain under the conditions used, we hypothesise that it may be transferrable to other strains belonging to the *P. putida* phylogenetic group. Strains belonging to this group display a GC content lower than those of *P. aeruginosa*, and differences in GC content are a known biological barrier for HGT [66].

Since secondary replicons are under strong pressure to undergo genomic reshuffling [64], the observed low nucleotide sequence identity between pJBCL41 and large *Pseudomonas* plasmids deposited in public databases might be expected. Even though pJBCL41 and pQBR103 are similar in size and functionalities, there is a high level of divergence between genes encoding related proteins. Indeed, it is rare to identify megaplasmids with a similar nucleotide sequence in strains belonging to different species within the same genus [6,52]. These results suggest that pJBCL41 and pQBR103 may share a common ancestor, but independent evolutionary trajectories have led to significant diversification among related genes.

The presence of different replicons suggests that pJBCL41 may have resulted from co-integration of distinct plasmid modules. The replication module defines plasmid copy number and plasmid survival in different hosts. Low copy-number plasmids are more frequently lost, due to random assortment at cell division [2,3] and extra stability modules, such as TA and partition systems, may be required to ensure that large plasmids such as pJBCL41 are maintained [48,67].

The DR flanking the 50 kb region in pJBCL41 and the related 59 kb region in the *P. aeruginosa* AR_0440 chromosome could reflect insertion of each region by transposition, possibly mediated by the intact transposase and resolvase of Tn*Pa40*. However, the size, complexity and differences between the internal parts of these related regions may be more consistent with initial insertion of a simple transposon followed by further insertions, deletions and rearrangements. A similar situation is seen in plasmid pCTX-M360, which carries a complete Tn*2* flanked by the 5 bp DR, and the highly-related pCTX-M3, in which the ends of Tn*2* are present in the same position but the central part of the transposon has undergone extensive rearrangements [68]. The identification of all or part of the 16,782 bp segment found within the 50 kb region in pJBCL41 in other locations also suggests that some of the genes it carries may encode advantageous functions, but this needs further analysis. Identification of other sequences related to parts of these 50 and 59 kb region segments may also shed light on how they have arisen and evolved.

In summary, we show that a hybrid Nanopore/Illumina approach is useful for producing contiguous assemblies and allowed full resolution of a carbapenemase-encoding *Pseudomonas* megaplasmid. The presence of this large plasmid may provide a selective advantage to the host cell. However, given their size and gene content, acquisition of these secondary replicons may pose a significant cost [69–71]. The high level of gene variation when compared to publicly available megaplasmids suggests that these secondary replicons frequently undergo gene loss and gain though HGT. The reduced purifying selection and the high prevalence of transposable elements frequently observed on megaplasmids may help to explain why these elements readily acquire foreign DNA [6,64,72]. In fact, mosaic plasmids such as pJBCL41 and the majority of megaplasmids have a high proportion of mobile genetic elements [73]. The identification of novel megaplasmids may shed light on the evolutionary effects of gene transfer and the selective forces driving antibiotic resistance.

## Supplementary Material

Supplemental Material
