# Supplementary material for: Combining sequencing approaches to fully resolve a carbapenemase-encoding megaplasmid in a Pseudomonas shirazica clinical strain
Source: Emerg Microbes Infect. 2019 Aug 5;8(1):1186–94. doi: 10.1080/22221751.2019.1648182 (PMC6713103; doi:10.1080/22221751.2019.1648182)
Supplement: Supplemental Material [file TEMI_A_1648182_SM1269.zip › S Tables 3.zip!/Table_S2.docx]

**Table S2**. Functional annotation of pJBCL41 and related megaplasmids. **A.** Absolute counts. **B.** Relative counts, corresponding to the fraction of each functional annotation calculated relative to the total annotated in a given plasmid.

**A**

| **Functional categories** | **AR439** | **P19E3** | **pBM413** | **pJB37** | **pJBCL41** | **pOZ176** | **pQBR103** | **pSY153** | **RW109** | **XWY-1** |
| --- | --- | --- | --- | --- | --- | --- | --- | --- | --- | --- |
| RNA processing and modification | 1 | 1 | 1 | 1 | 0 | 1 | 1 | 1 | 1 | 0 |
| Energy production and conversion | 1 | 12 | 0 | 2 | 4 | 4 | 1 | 0 | 15 | 8 |
| Cell cycle control and mitosis | 0 | 0 | 0 | 2 | 1 | 1 | 1 | 0 | 0 | 1 |
| Amino acid metabolism and transport | 2 | 2 | 2 | 3 | 4 | 4 | 2 | 2 | 37 | 3 |
| Nucleotide metabolism and transport | 0 | 0 | 0 | 0 | 0 | 0 | 0 | 0 | 2 | 1 |
| Carbohydrate metabolism and transport | 3 | 3 | 2 | 2 | 3 | 5 | 0 | 3 | 5 | 6 |
| Coenzyme metabolism | 3 | 4 | 4 | 3 | 3 | 3 | 2 | 9 | 5 | 3 |
| Lipid metabolism | 0 | 4 | 0 | 0 | 0 | 0 | 0 | 0 | 1 | 0 |
| Translation | 0 | 0 | 2 | 1 | 1 | 4 | 0 | 4 | 0 | 0 |
| Transcription | 13 | 18 | 13 | 22 | 15 | 29 | 13 | 16 | 33 | 22 |
| Replication and repair | 51 | 36 | 49 | 37 | 48 | 62 | 23 | 70 | 60 | 70 |
| Cell wall/membrane/envelope biogenesis | 11 | 8 | 4 | 6 | 3 | 3 | 5 | 5 | 11 | 10 |
| Cell motility | 3 | 3 | 3 | 3 | 5 | 3 | 4 | 3 | 3 | 0 |
| Post-translational modification, protein turnover and chaperone functions | 7 | 7 | 5 | 7 | 19 | 6 | 7 | 5 | 8 | 3 |
| Inorganic ion transport and metabolism | 8 | 15 | 5 | 12 | 6 | 10 | 4 | 9 | 14 | 12 |
| Secondary structure | 0 | 1 | 1 | 4 | 1 | 0 | 0 | 1 | 1 | 9 |
| Unknown function | 47 | 52 | 50 | 56 | 48 | 57 | 45 | 47 | 64 | 38 |
| Signal transduction | 20 | 21 | 17 | 20 | 23 | 19 | 15 | 17 | 22 | 10 |
| Intracellular trafficking and secretion | 5 | 5 | 5 | 17 | 6 | 17 | 6 | 6 | 4 | 13 |
| Annotated | 175 | 192 | 163 | 198 | 190 | 228 | 129 | 198 | 286 | 209 |

**B**

| **Functional categories** | **AR439** | **P19E3** | **pBM413** | **pJB37** | **pJBCL41** | **pOZ176** | **pQBR103** | **pSY153** | **RW109** | **XWY-1** |
| --- | --- | --- | --- | --- | --- | --- | --- | --- | --- | --- |
| RNA processing and modification | 0.6 | 0.5 | 0.6 | 0.5 | 0.0 | 0.4 | 0.8 | 0.5 | 0.3 | 0.0 |
| Energy production and conversion | 0.6 | 6.3 | 0.0 | 1.0 | 2.1 | 1.8 | 0.8 | 0.0 | 5.2 | 3.8 |
| Cell cycle control and mitosis | 0.0 | 0.0 | 0.0 | 1.0 | 0.5 | 0.4 | 0.8 | 0.0 | 0.0 | 0.5 |
| Amino acid metabolism and transport | 1.1 | 1.0 | 1.2 | 1.5 | 2.1 | 1.8 | 1.6 | 1.0 | 12.9 | 1.4 |
| Nucleotide metabolism and transport | 0.0 | 0.0 | 0.0 | 0.0 | 0.0 | 0.0 | 0.0 | 0.0 | 0.7 | 0.5 |
| Carbohydrate metabolism and transport | 1.7 | 1.6 | 1.2 | 1.0 | 1.6 | 2.2 | 0.0 | 1.5 | 1.7 | 2.9 |
| Coenzyme metabolism | 1.7 | 2.1 | 2.5 | 1.5 | 1.6 | 1.3 | 1.6 | 4.5 | 1.7 | 1.4 |
| Lipid metabolism | 0.0 | 2.1 | 0.0 | 0.0 | 0.0 | 0.0 | 0.0 | 0.0 | 0.3 | 0.0 |
| Translation | 0.0 | 0.0 | 1.2 | 0.5 | 0.5 | 1.8 | 0.0 | 2.0 | 0.0 | 0.0 |
| Transcription | 7.4 | 9.4 | 8.0 | 11.1 | 7.9 | 12.7 | 10.1 | 8.1 | 11.5 | 10.5 |
| Replication and repair | 29.1 | 18.8 | 30.1 | 18.7 | 25.3 | 27.2 | 17.8 | 35.4 | 21.0 | 33.5 |
| Cell wall/membrane/envelope biogenesis | 6.3 | 4.2 | 2.5 | 3.0 | 1.6 | 1.3 | 3.9 | 2.5 | 3.8 | 4.8 |
| Cell motility | 1.7 | 1.6 | 1.8 | 1.5 | 2.6 | 1.3 | 3.1 | 1.5 | 1.0 | 0.0 |
| Post-translational modification, protein turnover and chaperone functions | 4.0 | 3.6 | 3.1 | 3.5 | 10.0 | 2.6 | 5.4 | 2.5 | 2.8 | 1.4 |
| Inorganic ion transport and metabolism | 4.6 | 7.8 | 3.1 | 6.1 | 3.2 | 4.4 | 3.1 | 4.5 | 4.9 | 5.7 |
| Secondary structure | 0.0 | 0.5 | 0.6 | 2.0 | 0.5 | 0.0 | 0.0 | 0.5 | 0.3 | 4.3 |
| Unknown function | 26.9 | 27.1 | 30.7 | 28.3 | 25.3 | 25.0 | 34.9 | 23.7 | 22.4 | 18.2 |
| Signal transduction | 11.4 | 10.9 | 10.4 | 10.1 | 12.1 | 8.3 | 11.6 | 8.6 | 7.7 | 4.8 |
| Intracellular trafficking and secretion | 2.9 | 2.6 | 3.1 | 8.6 | 3.2 | 7.5 | 4.7 | 3.0 | 1.4 | 6.2 |
| Annotated | 32.0 | 32.1 | 30.4 | 33.2 | 31.3 | 36.7 | 23.1 | 34.1 | 44.1 | 45.9 |
| Un-Annotated | 68.0 | 67.9 | 69.6 | 66.8 | 68.8 | 63.3 | 76.9 | 65.9 | 55.9 | 54.1 |
